# Histopathological examination of the protective effect of intense exercise in apoptotic germ cell damage due to diabetes

**DOI:** 10.1590/acb381423

**Published:** 2023-04-21

**Authors:** Veysel Toprak, Senem Alkan Akalın, Ece Öcal, Yunus Çavuş, İlhan Özdemir

**Affiliations:** 1 Eyyübiye Education and Research Hospital – Department of Gynecology and Obstetrics – Şanlıurfa, Turkey.; 2 Private Medical Practice – Department of Gynecology and Obstetrics – Diyarbakir, Turkey.; 3 Antalya Research and Education Hospital – Department of Perinatology – Antalya, Turkey.; 4 Diyarbakir Memorial Hospital – Department of Gynecology and Obstetrics – Diyarbakir, Turkey; 5 Atatürk University – Faculty of Medicine – Department of Gynecology and Obstetrics – Erzurum, Turkey.

**Keywords:** Streptozotocin, Oxidative Stress, Testis, Cell Death

## Abstract

**Purpose:**

The aim of this study was to determine the protective and antioxidative effects of intensive exercise on streptozotocin (STZ)-induced testicular damage, apoptotic spermatognial cells death, and oxidative stress.

**Methods:**

36 male Sprague Dawley rats were divided into three groups: control, diabetes, and diabetes+intensive exercise (IE) groups. Testicular tissues were examined histopathologically and antioxidant enzymes, including catalase (CAT), superoxide dismutase (SOD), glutathione peroxidase (GPx), and malondialdehyde (MDA) activity, as well as serum testosterone level, were measured.

**Results:**

Seminiferous tubules and germ cells were found to be better in the testis tissue of the intense exercise group than in the diabetes group. Diabetes suppressed antioxidant enzymes CAT, SOD, GPx and testosterone levels were significantly decreased, and increased MDA level in the diabetic group compared to diabetes+IE group (p < 0.001). Following four weeks of treatment, intensive exercise improved the antioxidant defense, significantly decreased MDA activity, and increased testosterone levels in testicular tissue in the diabetic group compared to diabetes+IE group (p < 0.01).

**Conclusions:**

STZ-induced diabetes causes damage to the testis tissue. In order to prevent these damages, exercise practice has become very popular nowadays. In present study, our intensive exercise protocol, histological, and biochemical analysis of the effect of diabetes on the testicular tissues is shown.

## Introduction

Diabetes mellitus (DM) is one of the most common chronic diseases, affecting more than 100 million people in the world. It is a metabolic disease that causes long-term damage to many organs including the heart, eyes, kidneys, and testes, as well as the vascular system^1^. High glucose has been reported to cause oxidative stress and apoptosis in testicular cells, thus contributing to infertility^2^. DM is known to cause testicular cell death by inducing the apoptosis pathway. An experimental study has shown that cellular apoptosis is caused by testicular damage caused by hyperglycemia-induced oxidative stress^3^. It is known that streptozotocin (STZ)-induced diabetes has devastating effects on the male reproductive system. These injuries are due more to the development of diabetes than to the toxic effects of STZ or its derivatives^4^–^6^ In fact, elevation in blood glucose levels would lead to increased production of reactive oxygen species (ROS). Therefore, oxidative stress acts as a trigger, which results in decreased antioxidant defense and, ultimately, damage to the cell membrane and apoptosis in the testis. Moreover, increased ROS generation can bring about oxidative stress, endothelial injury, DNA damage, manifestation of necrosis, and apoptosis in germinal cells^6^^,^ 7.

The positive effects of exercise on human health are widely accepted today^8^. Physically fit men tend to have healthy sperm, but excessive exercise (particularly when combined with the use of illegal bodybuilding steroids and other drugs) can lower testosterone production and sperm count. It has been shown that exercise helps in weight control and contributes to the body with its preventive effect on the formation of stress hormones^9^. In order to better reveal the pathogenesis of diabetes and to evaluate the prevention and treatment options of the disease, experimental models of diabetes are created using various experimental animals^10^. Some studies have shown the positive effects of aerobic exercise, such as swimming, cycling, treadmill, walking, rowing, running, and jumping rope, based on different intensities on the improvement of type 2 DM^11^^,^ 12.

This experimental study aimed to reveal the role of intensive exercise on negative results such as infertility, blood glucose levels, seminiferous tubule diameters, and testicular weight change emerging in the testicular tissues of male rats with experimental diabetes induced by STZ. These apoptotic changes in the testicular tissue were planned to be revealed by histochemical and immunohistochemical staining. It also aimed to determine the protective and antioxidative effects of intensive exercise on STZ-induced testicular damage, apoptotic germ cell death, and oxidative stress.

## Methods

### Animals and experimental design

This study was approved by the Erzurum Ataturk University Ethics Committee. Thirty-six adult male Sprague Dawley rats with the same characteristics, ranging in weight from 25 to 300 g raised in Experimental Animal Research Unit of Ataturk University University. A total of three groups were formed in the experiment. The animals were kept under standard conditions and fed ad libitum. The sample size was chosen based on the literature on which the experimental exercise model was applied.

### Exclusion criteria

Subjects whose vital functions decreased very rapidly 3 days after STZ administration were not included in the study. Subjects who had difficulty in moving on the treadmill after the treatment was started, resisted, and did not run, and subjects who initially ran but later had energy problems due to diabetes and did not run were excluded from the study.

In addition, much more specific parameters were waived due to the limited budget. However, it should be said that diabetes complications can be overcome with more comprehensive and targeted studies to be done with exercise. at least this claim can be insisted on for infertility. The results confirm this.

### Experimental groups

Control group (n = 12): Citrate buffer (with a pH of 4.2; 0.1 mol L^–1^), given intraperitoneally as a single dose. Diabetes group (n = 12): STZ (dissolved in 0.1 mol L^–1^ citrate buffer with a pH of 4.2), and given intraperitoneally as a single dose of 40 mg/kg. Diabetes+IE group (n = 12): Group which started exercising 3 days prior to inducing diabetes with STZ (single dose of 40 mg/kg) and continued the intensive exercise (30 min at 30 m/min) 5 days a week until the end of the experiment.

STZ (Sigma Aldrich Chemicals, USA) was used to chemically induce diabetes. At 3 days after STZ injection, blood glucose levels were measured glucometer. The animals with blood glucose higher than 250 mg/dL were considered diabetic and included in the experiment. The control group received an equal volume of citrate buffer. At the end of the experiment, the testicular tissue was taken and stored at –20 °C for biochemical analysis. Testis samples were fixed in Bouin’s solution.

### Exercise procedure of rats

In the diabetes+IE group, exercise was started 3 days before and 3 days after the onset of diabetes. They received a single dose of 40 mg/kg STZ and continued the exercise until the end of the experiment. Using a motorized treadmill prepared for the rats, the intense exercise group was made run for 30 min at 30 m/min for 5 days a week on the treadmill.

### Biochemical analysis

The left testis of each animal was used for evaluation of antioxidant enzymes. The protein content of the supernatant was determined using Lowry method^13^. Catalase (CAT) activity was measured according to ultraviolet colorimetric method described by Aebi^14^, superoxide dismutase (SOD) activity was determined according to the colorimetric method of Martin Jr et al.^15^, glutathione peroxidase (GPx) activity was measured according to the method described by Ho et al.^16^, malondialdehyde (MDA) level was measured by spectrophotometry as per the method of Placer et al.^17^. Serum testosterone level was determined by enzyme-linked immunosorbent assay (ELISA) method using special rat kits (CusabioBiotech, Wuhan, China).

### Statistical analysis

Statistical analyzes were performed using Statistical Package for Social Sciences (SPSS), version 19.0 SPSS Inc. (Chicago, IL, USA). All these variables were given as mean ± standard deviation (SD). Significant differences between these three groups were analyzed with nonparametric Kruskal–Wallis test. Bilateral comparisons between groups were assessed with Mann–Whitney U test. These differences were considered significant when the p value was < 0.05.

### Histophatological investigation

For light microscopic examination, 10 seminiferous tubules were selected from the slides. Germinal epithelium and spermatogenic activity was examined in slides with 40× magnification. Scoring system was based on Johnsen scoring method as shown in Table 1 18.

**Table 1:** Johnsen scoring.

Score	Description
1	No cells in the seminiferous tubules
2	Only Sertoli cells
3	Only spermatogonium
4	Less than 5 spermatocytes
5	A lot of spermatocytes
6	Less than 10 spermatids present
7	Plenty of spermatids without differentiation spermatozoa
8	There are less than 10 spermatozoa in the lumen
9	There is a nonuniform appearance, leading to obliteration of the cell lumps in the lumen
10	There are multirow, abundant spermatozoa and tubules containing open lumen in the central

### Immunohistochemical investigation

Immunohistochemical reactions were performed according to the Avidin–Biotin complex (ABC) technique described. The procedure included the following steps: sections were incubated with specific mouse monoclonal antiproliferation cell nuclear antigen (PCNA) antibody (Cat No MS-106-B, Thermo LabVision, USA). The grade (ab7970, USA) was diluted 1:50 for 1 h at room temperature; sections were incubated with biotinylated antimouse immunoglobulin G (DAKO LSAB 2 Kit); sections were incubated with ABC (DAKO LSAB 2 Kit); peroxidase was detected by an aminoethylcarbazole substrate kit (AEC kit; Zymed Laboratories); nuclei stained with hematoxylin, and the sections were mounted on top of DAKO^19^.

For the proliferation index, 10 seminiferous tubule counts were made in each preparation. Germ cells stained with red were considered positive. Both stained and unstained germ cells were counted and the ratio of the total germ cell number to the stained cells was calculated as the PCNA index^20^.

*Terminal deoxynucleotide transferarasile dUTP nick end labeling (TUNEL) assay*

TUNEL method detects fragmentation in the DNA nucleus, was used in situ during apoptotic cell death, the apoptosis detection kit (TdT Fragel DNA Fragmentation Kit, Cat No QIA33, Calbiochem, USA). All reagents listed below and the manufacturer’s instructions below. They were then incubated for 20 min with 20 mg/mL of proteinase K and rinsed in tris-buffered saline. Endogenous peroxidase activity was inhibited by incubation 3% hydrogen peroxide. The sections were then incubated for 10 to 30 min with the equilibration buffer, then the terminal deoxynucleotidyl transferase enzyme in a humidified atmosphere at 37 °C for 90 min. Then, prewarmed working power for 10 min at room temperature was placed in stop/wash buffer and incubated with blocking buffer for 30 min. Each step was separated by extensive washings in tris-buffered saline. Labeling was elicited using diaminobenzidine tetrahydrochloride, contrast staining was carried out using methyl green and the sections were dehydrated, cleaned and mounted^19^^,^ 21.

Apoptotic index; TUNEL stained cell count and total germ cells count were calculated in 30 large magnification fields. It was then defined as the ratio of every 1,000 cells^20^.

## Results

### Blood glucose and serum testosterone levels

**Figure 1:**
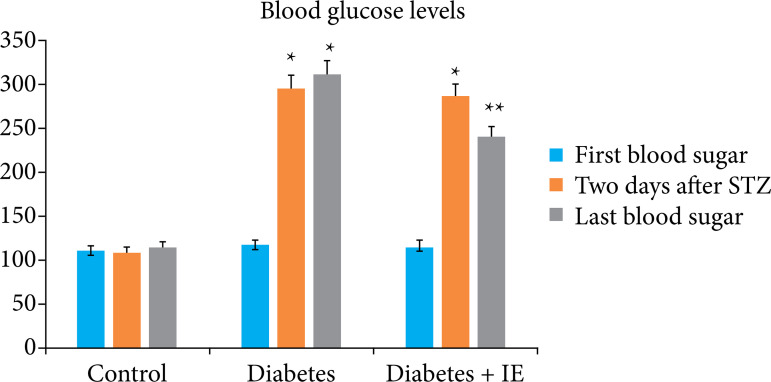
Blood glucose levels of control, diabetes, and diabetes +IE. *p < 0.0001 compared to the control group; **p *<* 0.05 compared to diabetes group.

Blood glucose values of all subjects were measured by glucometer before and after STZ administration. There was no statistically significant difference in blood glucose level of control group. Diabetes induction resulted in a significant reduction in serum testosterone levels of diabetic rats compared to the control rats. Treatment with intensive exercise for 4 weeks could significantly recover the serum testosterone changes to the normal levels expected other groups rat (Fig. 1, Table 2).

**Table 2:** Effect of intensive exercise on blood glucose and serum testosterone levels in the rat testis after STZ-induced diabetes.

	Control (n = 12)	Diabetes (n = 12)	Diabetes+IE (n = 12)
Testosterone (ng/mL)	3.60 ± 0.40	1.80 ± 0.08^*^	2.60 ± 0.56^**^
Glucose (mg/dL)	117.00 ± 0.70	322.00 ± 13.17^*^	275.00 ± 12.84^***^

Values were expressed as mean ± SD.

* p<0.001 compared to control group.

** p<0.05 compared to diabetes group.

*** p < 0.01 compared to diabetes+IE group.

### Antioxidant findings

The activities of antioxidant enzymes in testicular tissue, including CAT, SOD, and GPx, was significantly lower (p < 0.05) than those of the control group. STZ-induced diabetes resulted in significant increases of MDA levels in the testis tissues when compared with the control group. Intensive exercise effectively improved the activities of SOD and GPx toward normal levels. The activity of CAT was increased exercise group, albeit not to the levels in the control group. The levels of MDA in exercise groups were significantly (p < 0.05) lower than those of diabetic rats (Table 3).

**Table 3:** Levels of testis CAT, SOD, GPx and MDA in all groups.

Groups	CAT (U/mL)	SOD (U/mL)	GPx (U/mL)	MDA (nmol/mL)
Control	49.82 ± 5.12	46.23 ± 7.42	386.21 ± 34.54	2.82 ± 0.60
Diabetes	12.64 ± 1.20^*^	18.78 ± 3.40^*^	257.45 ± 23.20^*^	7.25 ± 2.20^*^
Diabetes+IE	33.50 ± 5.45^**^	29.32 ± 3.74^**^	348.20 ± 30.82^**^	3.92 ± 1.26^**^

Values were expressed as mean ± SD.

* p < 0.001 compared to diabetes group.

** p < 0.01 compared to diabetes+IE group.

### Testicular weights

**Figure 2:**
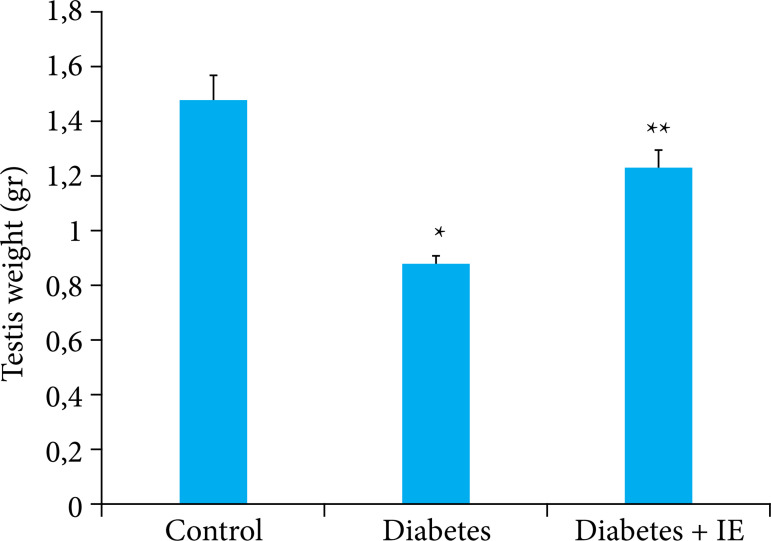
Testes weights of control, diabetes, and diabetes+IE groups. *p < 0.001 compared to diabetes group; **p < 0.01 compared to diabetes+IE group.

When compared with the control group, it was observed that there was a significant decrease in testicular weights in all test groups (Fig. 2).

### Diameter of seminiferous tubules

**Figure 3:**
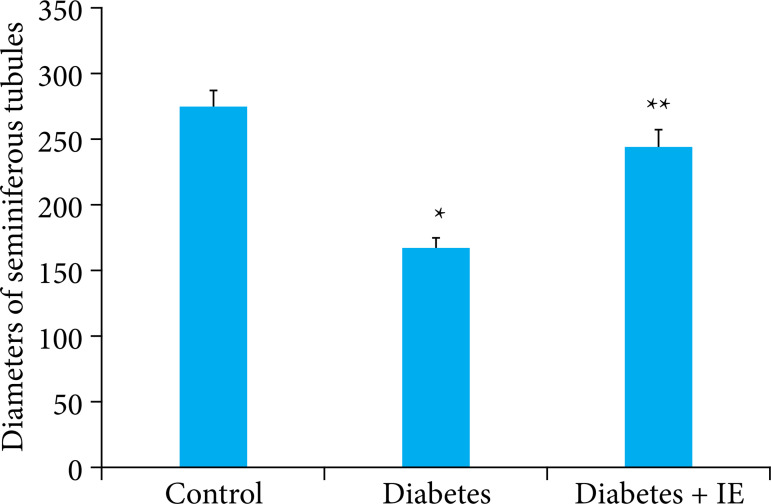
Seminiferous tubules diameters belonging to control, diabetes, and diabetes+IE groups. *p < 0.001 compared to diabetes group; **p < 0.05 compared to diabetes+IE group.

Changes in seminiferous tubules due to diabetes were shown by the measurement of tubule diameters. When the experimental groups were compared with the control group, seminiferous tubule diameters of the diabetes group showed a statistically significant decrease (p < 0.001); The seminiferous tubule diameters of the diabetic group were significantly lower than those of the intensive exercise group (p < 0.05) (Fig. 3).

### H&E findings

**Figure 4:**
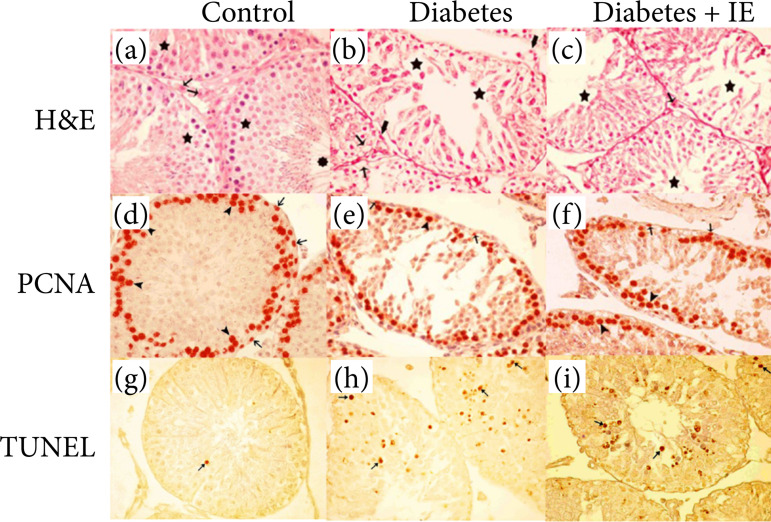
Hematoxylin and eosin staining (H&E) (a-c), PCNA immune staining (d-f) and TUNEL assay results (g-i) of experimental groups.

The testicular tissue sections obtained from the subjects belonging to the control group and stained with H&E were normally after exam (Fig. 4a). Diabetes induced the morphological alterations such as reduction in the size of the seminiferous tubules, degeneration and vacuolization in spermatogonia, spermatocytes, and spermatids. Atrophic changes were observed in the testicular tissues of the diabetes group in most of the seminiferous tubules, and the cells were separated from the basement membrane and discharged into the lumen. It was determined that the germinal epithelial layer appeared to have large vacuoles due to the degeneration of Sertoli cells located near the basal part of the tubules in the diabetes group (Fig. 4b). In the testicular tissue of the diabetes group, atrophic changes observed in most seminiferous tubules and the shape and size differences between the tubules were observed to be less in intensive exercise, and that the cells of the spermatogenic cell series including spermatozoa were preserved. In addition, the presence of Sertoli cell degeneration was detected. The damage in the Leydig cells in the interstitial area was also reduced compared to the diabetes group. The number of spermatogenic cells in the diabetes+IE group was observed to be increased compared to the diabetes group (Table 4, Fig. 4c).

**Table 4:** Counting of spermatogenic cells for each group.

Spermatogenic cells	Control (n = 12)	Diabetes (n = 12)	Diabetes+IE (n =12)
Spermatogonia	16.21 ± 0.84	8.23 ± 0.46^a^	13.04 ± 0.58^b^
Spermatocytes	118.19 ± 2.10	33.74 ± 2.87^c^	91.20 ± 9.54^d^
Spermatids	127.13 ± 4.12	35.18 ± 3.72^c^	96.82 ± 11.33^d^
Sertoli cells	14.91 ± 0.62	6.86 ± 0.71^a^	11.75. ± 0.55^b^

Values were expressed as mean ± SD.

(a) p < 0.01 compared to control group;

(b) p < 0.05 compared to diabetic group;

(c) p < 0.001 compared to control group;

(d) p < 0.001 compared to diabetic group.

### PCNA findings

**Figure 5:**
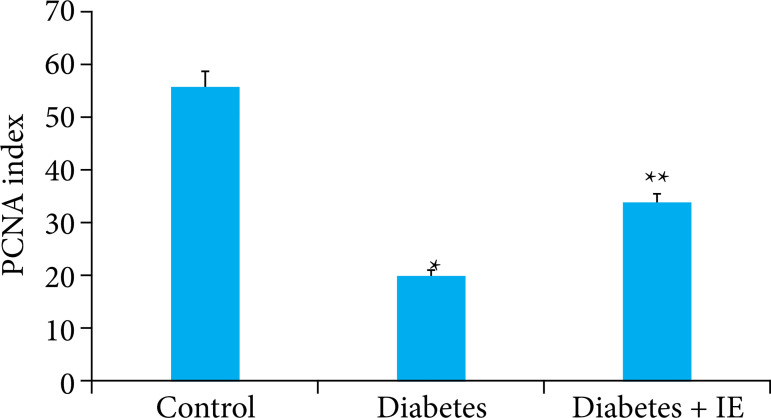
PCNA index of control, diabetes, and diabetes+IE groups. *p < 0.001 compared to diabetes group; **p < 0.01 compared to diabetes+IE group.

When PCNA immunostained slides were examined, differences were observed between the groups in terms of positive cell frequency. In the control group, many spermatocytes and spermatogonium in the seminiferous tubules showed PCNA positive reaction (Fig. 4d), whereas in the diabetes group it was less than the control group (Fig. 4e). In the diabetes+IE group, it was observed that the number of PCNA positive cells increased compared to the diabetes group (Fig. 4f). There was a statistically significant difference in PCNA index values between control, diabetes and diabetes+IE groups (Fig. 5).

### TUNEL findings

**Figure 6:**
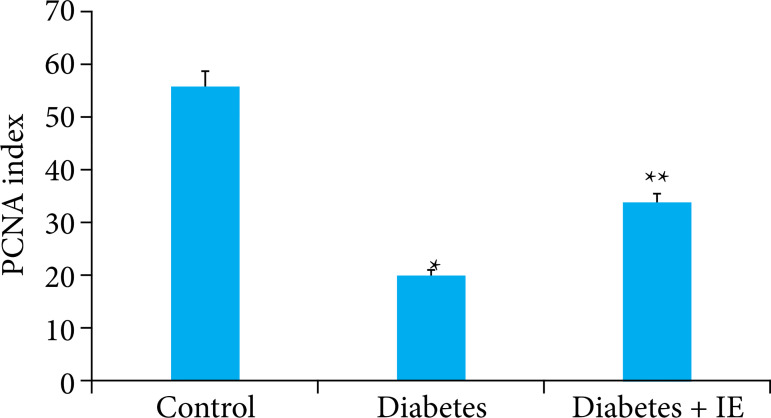
Apoptotic index values of control, diabetes, and diabetes+IE groups. *p < 0.001 compared to diabetes group. **p < 0.01 compared to diabetes+IE group.

Apoptotic cells were determined by TUNEL staining in all groups. A statistically significant difference was found between control, diabetes, and intense exercise groups in apoptotic index values. Very few TUNEL positive cells were observed in the control group (Fig. 4g). However, the number of TUNEL positive cells increased significantly in the diabetes group (Fig. 4h). It was found that the number of apoptotic cells decreased in the diabetes+IE group compared to the diabetes group (Figs. 4i, 6).

## Discussion

The present study shows that intensive exercise in a positive way regulates oxidative stress and alleviates testicular damage and apoptotic germ cell death in STZ-induced diabetes in adult male rats. STZ-induced diabetes resulted in significant rise of MDA levels and caused important decrease of the testosterone levels and the SOD and GPx antioxidant enzymes activities in the testis tissue. Whole changes were improved by the intensive exercise. The reproductive dysfunction in diabetes is primarily either due to the oxidative stress generated by free radicals or the instability between the prooxidant and antioxidant status^22^.

In inducing experimental diabetes, chemicals such as STZ and alloxan are mostly use^23^. STZ, one of the substances used in the induction of experimental diabetes, demonstrates its diabetic effect by destroying β cells in the pancreas. STZ was shown to disrupt oxidation of glucose, and that biosynthesis and release of insulin was reduced^24^. The damage caused by diabetes is due to the formation of oxidative stress in the tissues^25^^,^ 26. It is known that STZ-induced diabetes has devastating effects on the male reproductive system. These injuries are due more to the development of diabetes, than to the toxic effects of STZ or its derivatives^27^. In fact, elevation in blood glucose levels would lead to increased production of ROS. Therefore, oxidative stress acts as a trigger, which results in decreased antioxidant defense and, ultimately, damage to the cell membrane and apoptosis in the testis. STZ-induced diabetes resulted in significant increases of MDA levels and induced significant reduction of the testosterone levels and the SOD and GPx antioxidant enzymes activities in the testis. All these changes were ameliorated by the treatment of exercise. The reproductive dysfunction in diabetes is mainly either due to the oxidative stress generated by the free radicals or the imbalance between the prooxidant and antioxidant status^28^. In the present study, intensive exercise produced a significant decrease in lipid peroxidation compared with the diabetic group. In the diabetes+IE group, the activities of GPx, CAT and SOD were increased compared with the diabetic group.

Increased peroxidation of lipid due to varicocele leads to excessive production of free radicals such as hydroxyl, superoxide, and peroxyl, resulting in the decreased endogenous antioxidants activity of the including GPx and SOD enzymes. Therefore, varicocele can disturb the equilibrium of pro-oxidant and antioxidant elements of affected testis. Similarly, ROS, which occurs as a complication of diabetes, triggers infertility by causing testicular damage. According to other study, the extreme generation of ROS in diabetes-affected testicles may lead to the DNA and protein damage leading to the apoptosis^29^. Oxidative stress plays a role in the development of diabetic complications^30^. The levels of antioxidants such as SOD and GPx were significantly reduced in the subjects whose stress balance was disturbed by the experimental varicocele model. In this study, both antioxidant defense elements (SOD, CAT, GPx) decreased and MDA due to oxidants increased. Although damage occurred through different mechanisms, the result was the same. Oxidative stress accelerated the apoptotic process. This study showed that STZ-induced diabetes increased apoptosis in seminiferous tubules of rats and mice. When only the apoptotic index values obtained from the evaluation of cells, whose apoptotic nuclei were stained as a result of TUNEL staining, were compared, a statistically significant difference was found between control, diabetes and diabetes+IE groups. These findings are similar to the findings of a previous study^29^.

Oxidative stress plays a role in the development of diabetic complications^31^^,^ 32. Free radicals caused DNA damage and as a result of this damage, amount of free radical can be quantitatively determined by measuring MDA level. Certain enzymes play an important role in antioxidant defense to maintain viable reproductive ability; a protective mechanism against oxidative stress is of importance. These enzymes include SOD, GPx, glutathione reductase and CAT, which convert free radicals or reactive oxygen intermediates to nonradical products. SOD and GPx are major enzymes that scavenge harmful ROS in male reproductive organs^33^. Results of present study show that intensive exercise effectively improved the activities of SOD and GPx toward normal levels. The activity of CAT was increased in diabetes+IE group, albeit not to the levels in the control group. The levels of MDA in diabetes+IE group were significantly lower than those of diabetic rats (p < 0.05) (Table 3).

According to results of the present study, the size of the seminiferous tubules was strongly reduced when the morphology of the epithelium was severely impaired. The seminiferous tubule structure in the diabetic rats was found to be disrupted, and there was a considerable decrease in the spermatogenic cell line. Also in diabetic rats, atrophy of the tubules with varying degree of spermatogenetic arrest was detected. These observations agree with previous study^34^. In this study, Johnsen’s scores of intensive exercise with diabetic rats were higher and increased seminiferous tubule diameters when compared to diabetic group (Table 1).

Exercise, in addition to diet regimens and medications, has long been considered as one of the three main components of diabetes treatment. Because of its low cost and nonpharmacological properties, the interest in exercise among therapeutic approaches is increasing^35^. It has been reported that a person with diabetes who regularly exercise during their lifetime is protected against some of the changes in testicular tissue due to diabetes. Similarly, it was investigated histologically that the damage caused by diabetes in rats’ testes was corrected by exercise^36^^,^ 37.

In this study, the seminiferous tubules of the control group rats were observed as normal and healthy, while histopathological changes were observed in the diabetes group. Degenerative and sclerotic changes were evident in the testes of diabetic rats, but these changes were less in the diabetes+IE group. Thus, it is thought that exercise may have a healing effect against these changes in the testicular tissue.

PCNA expressed in spermatogonia and early-phase primary spermatocytes in all stages of the seminiferous tubules has been used previously to characterize testis tissues. PCNA-positive cells were detected in spermatogonia and early-phase primary spermatocytes, regardless of the stage of the seminiferous tubules^38^. The decrease of PCNA in testicular germ cells indicates the reduction of proliferative activity and spermatogenesis. Salama et al.^39^ examined the impact of aging and noninsulin-dependent DM on the expression of the PCNA in rat testicular tissue, and they found that older animals showed the fewest number of immunostained basal germ cells in the seminiferous tubuli. Also in diabetic rats, the PCNA index was decreased in the diabetic groups. This observation agrees with previous study^36^.

This study showed that STZ-induced diabetes increased apoptosis in seminiferous tubules of rats and mice. When only the apoptotic index values obtained from the evaluation of cells, whose apoptotic nuclei were stained as a result of TUNEL staining, were compared, a statistically significant difference was found between control, diabetes and diabetes+IE groups^2^^,^ 40.

## Conclusion

Histological changes due to complications of diabetes were demonstrated by TUNEL and PCNA methods in testicular tissues of diabetes-induced rats by STZ. Thus, this study aimed to demonstrate whether exercise has a therapeutic effect on abnormal spermatogenesis and loss of germ cells in the testicular tissue of rats with diabetes.

This research indicates that exercise improves diabetes-related testicular dysfunction and histopathological changes. Intensive exercise, downregulated the levels of ROS, reduced the number of TUNEL-positive cells, and increased PCNA activity in the testis of diabetic rats. Intensive exercise has been shown to be effective in preventing diabetes-related damage on testicular tissues.

## Data Availability

All data sets were generated or analyzed in the current study
